# Occupational Radiation Exposure during Endoscopic Retrograde Cholangiopancreatography and Usefulness of Radiation Protective Curtains

**DOI:** 10.1155/2014/926876

**Published:** 2014-11-13

**Authors:** Tomoyuki Minami, Tamito Sasaki, Masahiro Serikawa, Michihiro Kamigaki, Masanobu Yukutake, Takashi Ishigaki, Yasutaka Ishii, Teruo Mouri, Satoshi Yoshimi, Akinori Shimizu, Tomofumi Tsuboi, Keisuke Kurihara, Yumiko Tatsukawa, Eisuke Miyaki, Kazuaki Chayama

**Affiliations:** Department of Gastroenterology and Metabolism, Hiroshima University, 1-2-3 Kasumi, Minami-ku, Hiroshima 734-8551, Japan

## Abstract

*Objective*. To evaluate the effectiveness of radiation protective curtains in reducing the occupational radiation exposure of medical personnel. *Methods*. We studied medical staff members who had assisted in 80 consecutive therapeutic endoscopic retrograde cholangiopancreatography (ERCP) procedures. Use of radiation protective curtains mounted to the X-ray tube was determined randomly for each procedure, and radiation doses were measured with electronic pocket dosimeters placed outside the protective apron. *Results*. When protective curtains were not used, the mean radiation doses to endoscopists, first assistants, second assistants, and nurses were 340.9, 27.5, 45.3, and 33.1 *µ*Sv, respectively; doses decreased to 42.6, 4.2, 13.1, and 10.6 *µ*Sv, respectively, when protective curtains were used (*P* < 0.01). When the patient had to be restrained during ERCP (*n* = 8), the radiation dose to second assistants without protective curtains increased by a factor of 9.95 (*P* < 0.01) relative to cases in which restraint was not required. *Conclusions*. During ERCP, not only endoscopists, but also assistants and nurses were exposed to high doses of radiation. Radiation exposure to staff members during ERCP was reduced with the use of protective curtains.

## 1. Introduction

Techniques related to endoscopic retrograde cholangiopancreatography (ERCP) and endoscopic ultrasonography (EUS) are becoming more widely used in the field of gastrointestinal endoscopy [1]. Although such advances have allowed patients to receive minimally invasive treatments, they take longer to perform and, consequently, expose both patients and medical staff members (occupational exposure) to higher doses of radiation. There is no established radiation exposure threshold for patients, because exposure is permitted as long as the benefits of the examination or treatment outweigh the risks of exposure. In contrast, there are strict annual permissible doses for medical personnel, because they receive no benefit from radiation exposure. Reducing the radiation exposure of patients and medical personnel is an important issue [2–6].

It is already known that scatter radiation with overhead-tube fluoroscopic equipment is higher than that with undercouch-tube equipment. Lens injuries induced in nonoptimized interventional radiology laboratories have been reported [7]. However, overhead-tube equipment is still often used in endoscopic procedures. Equipment such as radiation protective clothing and eyewear is used to prevent exposure to scattered radiation from patients, which is the main source of occupational radiation exposure for health care providers. However, these types of protective equipment do not cover some parts of the body, and it is very important to protect the entire body. In an experimental and clinical study, Kurihara et al. found that using a protective lead shield mounted to the X-ray tube was effective in reducing the radiation dose to staff members [8]. However, it is not yet known how effective a protective lead shield would be for protection in clinical practice. In this study, we assessed the level of occupational radiation exposure during ERCP and measured the radiation doses to staff members when a protective lead shield was used and was not used during ERCP to assess the effectiveness of the shield.

## 2. Materials and Methods

### 2.1. Subjects

The subjects were medical staff members who had assisted in 80 consecutive therapeutic ERCP procedures performed at our hospital between August and October 2012. ERCP-related procedures under this study contained biliary drainage, pancreatic duct drainage, and lithotripsy, but balloon enteroscope-assisted ERCP which should need longer procedure time was excluded. The study design was prospective. Use of a protective lead shield was determined randomly for each ERCP procedure, and doses of scattered radiation to the medical staff members were measured. The randomization was performed using the shield envelope method. Consent regarding the random assignment of protective equipment and measurement of radiation doses was obtained in writing from the medical staff members.

### 2.2. X-Ray Fluoroscopy Generator and Protective Lead Shield

**Figure 1:**
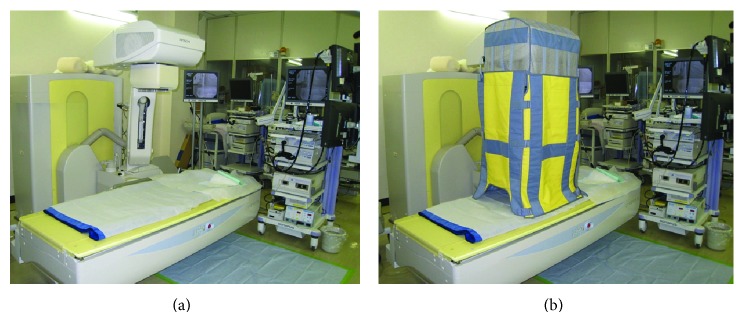
X-ray fluoroscopy generator and protective lead shield. (a) Without protective lead shield. (b) With protective lead shield.

The X-ray fluoroscopy generator was a CUREVISTA (Hitachi Medical Corporation, Japan). Examinations were performed at 25 × 25 cm and 30 pulses/second (Figure 1). The protective lead shield was a Hagoromo X-ray protective curtain (Maeda & Co., Ltd, Tokyo, Japan) that attaches to the X-ray tube, which was modified with the newly developed protect shield created by Itoi and his colleague [9]. The hood of the protective equipment was placed over the X-ray tube and attached to four shielding sheets that hang down to the surface of the operating table. The shielding sheets are made of a lead equivalent that is 0.25 mm thick on the endoscopist's side and 0.125 mm thick on the other three sides.

### 2.3. Exposure Measurements

**Figure 2:**
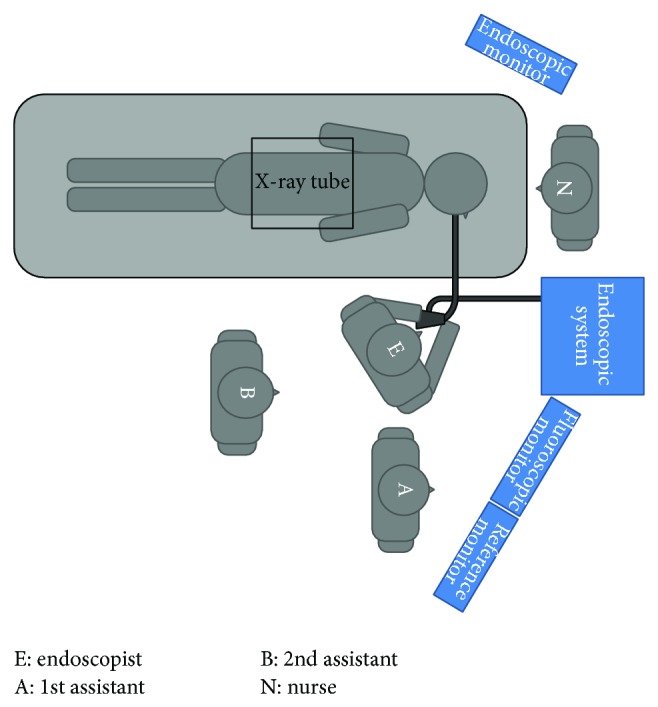
Positions of staff members relative to the patient and X-ray tube during the ERCP procedure. E: Endoscopist, A: 1st assistant, B: 2nd assistant, N: nurse, and ERCP: endoscopic retrograde cholangiopancreatography.

A four-person team of medical staff members comprising three doctors and one nurse performed each ERCP procedure (Figure 2). For all procedures, regardless of whether protective curtains were used, a PDM-117 semiconductor-type electronic pocket dosimeter (Hitachi Aloka Medical Co., Ltd., Tokyo, Japan) was affixed to the left anterior chest area of the outside of each staff member's protective lead apron, and radiation doses (in *μ*Sv) were measured. Patients were sedated with 0.05–0.23 mg/kg of midazolam during the ERCP procedures. If the patient moved, the medical staff members manually restrained the patient. The cases that the patients were restrained by the medical staff members were made into “movement (+).” The patient's skin entrance dose (in mGy), as calculated by the fluoroscopy generator with the numerical dose determination (NDD) method [10], was used to represent the radiation dose from the X-ray fluoroscopy generator.

### 2.4. Statistical Analysis

Statistical analysis was performed with JMP 9 (SAS Institute, Inc., Cary, NC, USA). Wilcoxon tests or *χ* ^2^ tests were performed, and *P* < 0.05 was considered significant.

## 3. Results

### 3.1. Radiation Doses to Patients and Medical Staff Members

For the 40 ERCP procedures (27 male patients, 13 female patients) in which protective curtains were not used, the mean procedure time was 26.8 min, and the mean skin entrance dose was 265.4 mGy. For the 40 ERCP procedures (20 male patients, 20 female patients) in which protective curtains were used, the mean procedure time was 28.5 min, and the mean skin entrance dose was 245.1 mGy (Table 1).

The mean radiation doses during ERCP when protective curtains were not used were 340.9, 27.5, 45.3, and 33.1 *μ*Sv for endoscopists, first assistants, second assistants, and nurses, respectively (Table 2).

For cases in which the patient's movement had to be restrained during ERCP (*n* = 8), the mean radiation doses to the medical staff members, in the aforementioned order, were 375.3, 29.0, 161.4, and 63.5 *μ*Sv, respectively. For cases in which restraint was not required (*n* = 32), these mean radiation doses were 332.3, 27.1, 16.2, and 25.5 *μ*Sv, respectively. Additionally, for cases in which the patient had to be restrained, the radiation doses to second assistants and nurses when protective curtains were not used increased by factors of 9.95 (*P* < 0.001) (Table 3).

### 3.2. Radiation Protective Effects of Protective Curtains

When protective curtains were used, the mean radiation doses to medical staff members were 42.6, 4.2, 13.1, and 10.6 *μ*Sv for endoscopists, first assistants, second assistants, and nurses, respectively. Use of protective curtains decreased the aforementioned doses by 87.5%, 84.7%, 71.1%, and 68.0% (*P* < 0.01) (Table 2). The mean radiation doses during the 12 procedures in which protective curtains was used and body movement had to be restrained, in the same order as above, were 78.7, 6.8, 35.7, and 20.8 *μ*Sv (Table 4).

## 4. Discussion

The 1990 Recommendations of the International Commission on Radiological Protection (ICRP) set safety standards for radiation exposure of 500 mSv/year for skin and 150 mSv/year for eyes [11]. Exposure of medical staff members to radiation is a critical problem, but there has been very little discussion about the topic. It is well recognized among diagnostic and interventional radiologists that scatter radiation with overhead-tube fluoroscopic equipment is considerably higher than that with undercouch-tube equipment. Use of overhead-tube equipment is not recommended for interventional procedures that require a long fluoroscopic time [12]. However, overhead-tube equipment is still used frequently in endoscopic procedures. Consequently, the use of effective radiation protectors is important to avoid radiation injuries for medical staff members engaging in ERCP.

In this study, we assessed the effectiveness of protective curtains in reducing radiation exposure in clinical practice. We measured radiation from the outside of the protective lead apron, because, in a preliminary study, we found that radiation inside the apron was below detectable levels in a small number of cases. Occupational exposure for all medical staff members decreased when a protective curtain was used; the radiation dose to the endoscopist during one ERCP procedure decreased by 87.5%, and the radiation doses to other staff members decreased by 68–85%. Use of protective curtains reduced radiation in the air throughout the entire operating room, which likely contributed to the reduction in the radiation exposure of all medical staff members at the whole-body level. To ensure the safety of medical staff members, it is important to use not only standard protective equipment, such as radiation protective clothing and eyewear, but also protective curtains.

In this study, we measured radiation doses from each ERCP procedure with high-sensitivity semiconductor-type dosimeters. We found that the radiation dose to endoscopists was approximately ten times greater than that to any other member of the medical staff, which indicates that radiation is a much more substantial issue for endoscopists. Buls et al. and Sulieman et al. similarly found that the radiation dose to endoscopists during ERCP is the highest among all staff members, which is consistent with our results [2, 13]. The endoscopist cannot keep a distance from the patient, and thus multiple levels of protection, consisting of not only protective clothing and eyewear, but also protective curtains, are required to protect the endoscopist from radiation exposure.

In this study, we measured the radiation doses to staff members during each procedure and found that a patient's movement during the examination greatly influenced these doses. When a patient moved substantially, the radiation dose to second assistants increased by a factor of ten. Substantial patient movement can interfere with the ERCP procedure, and thus a staff member must manually restrain the patient in such cases. This task is typically performed by a second assistant, and it is difficult to keep a distance from the patient when doing so, which is likely why their radiation dose was higher. Although few cases require this kind of restraint, the patient's movement leads to a considerable increase in radiation exposure. Therefore, to reduce exposure, it is important to create an environment in which the staff members are not exposed to scattered radiation, even when close to the patient.

However, one disadvantage of using protective curtains is that it blocks the surgical team's view of the patient. It is essential to monitor breathing for safety management during ERCP, because patients are sedated, and thus it may be dangerous if the staff cannot visually confirm chest movements. However, it is also possible to monitor breathing with devices such as a pulse oximeter or a capnometer, and we did not experience any issues in this study. In addition, the protective curtains used in this study weighed approximately 8 kg and could potentially damage the fluoroscopy generator if left in place for a long time. Although this study only lasted two months, we have been using the protective curtains continually for approximately a year since the end of the study, and it has not caused any issue with the fluoroscopy generator. In addition, about the patient's exposure, there was no significant difference by the curtain use both in the preliminary phantom experiment and this present study.

It is essential to ensure the health and safety of medical staff members while providing patients with a higher standard of care. The radiation protective X-ray tube attachment that we tested in this study reduced the radiation dose to all medical staff and should become more widely used in the future.

## 5. Conclusion

Not only endoscopists, but also assistants and nurses are exposed to a high dose of radiation during ERCP, which is why appropriate measures must be taken. Use of protective curtains reduced the radiation dose to all medical staff members by shielding the entire operating room from scattered radiation and thus was very effective in reducing occupational radiation exposure.

## Figures and Tables

**Table 1:** ERCP conditions.

X-ray fluoroscopy generator	CUREVISTA		*P* value
	Protective curtains (−)	Protective curtains (+)	
Procedure time, min	27.6 ± 15.0	27.9 ± 14.3	0.85
Fluoroscopy time, min	14.5 ± 10.0	13.0 ± 6.7	0.81
Number of image acquisitions, *n*	14.7 ± 7.5	17.1 ± 7.5	0.12
Tube voltage, Kv	84.4 ± 3.8	85.5 ± 4.2	0.59
Entrance dose to patients, mGy^*^	265.4 ± 247.3	245.0 ± 154.7	0.62

ERCP: endoscopic retrograde cholangiopancreatography. Values are presented as mean ± standard deviation.

^*^Numerical dose determination.

**Table 2:** Occupational radiation dose to medical staff members (*µ*Sv).

	Protective curtains (−)	Protective curtains (+)	*P* value
Endoscopist	340.9 ± 313.9	42.6 ± 54.7	<0.001
1st assistant	27.5 ± 48.5	4.2 ± 5.4	<0.001
2nd assistant	45.3 ± 81.3	13.1 ± 19.9	<0.001
Nurse	33.1 ± 43.7	10.6 ± 15.4	0.001

Values are presented as mean ± standard deviation.

**Table 3:** Occupational radiation dose to each medical staff member with or without patient movement (without the use of protective curtains) (*µ*Sv).

	Patient movement (−)	Patient movement (+)	*P* value
Endoscopist	332.3 ± 336.0	375.3 ± 217.3	0.367
1st assistant	27.1 ± 52.4	29.0 ± 31.0	0.046
2nd assistant	16.2 ± 15.6	161.4 ± 128.4	<0.01
Nurse	25.5 ± 34.2	63.5 ± 64.5	0.013

Values are presented as mean ± standard deviation.

**Table 4:** Occupational radiation dose to each medical staff member with or without patient movement (with the use of protective curtains) (*µ*Sv).

	Patient movement (−)	Patient movement (+)	*P* value
Endoscopist	27.1 ± 21.3	78.7 ± 86.4	<0.01
1st assistant	3.1 ± 4.4	6.8 ± 6.7	0.044
2nd assistant	3.4 ± 2.9	35.7 ± 24.1	<0.01
Nurse	6.2 ± 6.0	20.8 ± 24.2	0.033

Values are presented as mean ± standard deviation.
